# Induction of nerve growth factor expression and release by mechanical and inflammatory stimuli in chondrocytes: possible involvement in osteoarthritis pain

**DOI:** 10.1186/ar4443

**Published:** 2014-01-20

**Authors:** Emilie Pecchi, Sabrina Priam, Marjolaine Gosset, Audrey Pigenet, Laure Sudre, Marie-Charlotte Laiguillon, Francis Berenbaum, Xavier Houard

**Affiliations:** 1 INSERM UMRS938, UPMC, Univ Paris 06, 7 quai Saint-Bernard, Paris F-75252, Cedex 5, France; 2 Department of Rheumatology, Inflammation-Immunopathology-Biotherapy Department (DHU i2B), AP-HP Saint-Antoine Hospital, 184, rue du Faubourg Saint-Antoine, F-75012 Paris, France; 3 Département de Rhumatologie, Hôpital Saint-Antoine, 184, rue du Faubourg Saint-Antoine, F-75012 Paris, France

## Abstract

**Introduction:**

Nerve growth factor (NGF) level is increased in osteoarthritis (OA) joints and is involved in pain associated with OA. Stimuli responsible for NGF stimulation in chondrocytes are unknown. We investigated whether mechanical stress and proinflammatory cytokines may influence NGF synthesis by chondrocytes.

**Methods:**

Primary cultures of human OA chondrocytes, newborn mouse articular chondrocytes or cartilage explants were stimulated by increasing amounts of IL-1β, prostaglandin E_2_ (PGE_2_), visfatin/nicotinamide phosphoribosyltransferase (NAMPT) or by cyclic mechanical compression (0.5 Hz, 1 MPa). Before stimulation, chondrocytes were pretreated with indomethacin, Apo866, a specific inhibitor of NAMPT enzymatic activity, or transfected by siRNA targeting visfatin/NAMPT. mRNA NGF levels were assessed by real-time quantitative PCR and NGF released into media was determined by ELISA.

**Results:**

Unstimulated human and mouse articular chondrocytes expressed low levels of NGF (19.2 ± 8.7 pg/mL, 13.5 ± 1.0 pg/mL and 4.4 ± 0.8 pg/mL/mg tissue for human and mouse articular chondrocytes and costal explants, respectively). Mechanical stress induced NGF release in conditioned media. When stimulated by IL-1β or visfatin/NAMPT, a proinflammatory adipokine produced by chondocytes in response to IL-1β, a dose-dependent increase in NGF mRNA expression and NGF release in both human and mouse chondrocyte conditioned media was observed. Visfatin/NAMPT is also an intracellular enzyme acting as the rate-limiting enzyme of the generation of NAD. The expression of NGF induced by visfatin/NAMPT was inhibited by Apo866, whereas IL-1β-mediated NGF expression was not modified by siRNA targeting visfatin/NAMPT. Interestingly, PGE_2_, which is produced by chondrocytes in response to IL-1β and visfatin/NAMPT, did not stimulate NGF production. Consistently, indomethacin, a cyclooxygenase inhibitor, did not counteract IL-1β-induced NGF production.

**Conclusions:**

These results show that mechanical stress, IL-1β and extracellular visfatin/NAMPT, all stimulated the expression and release of NGF by chondrocytes and thus suggest that the overexpression of visfatin/NAMPT and IL-1β in the OA joint and the increased mechanical loading of cartilage may mediate OA pain via the stimulation of NGF expression and release by chondrocytes.

## Introduction

Osteoarthritis (OA) is a chronic and age-related joint disease leading to cartilage destruction. Whereas the mechanisms by which this degradation happens are more and more understood, the reasons why an OA joint is painful are quite mysterious. For a same degree of cartilage degradation, some patients have symptoms and others have not. Recently, novel pharmacological molecules, belonging to the anti-nerve growth factor (NGF) family, have shown a dramatic effect on OA symptoms, much more efficacious than non-steroidal anti-inflammatory drugs (NSAIDs), the usual treatment for symptomatic OA [1-4]. Unfortunately, all clinical trials were halted in 2011 due to an unexpected increase in the number of total joint prosthesis in the active compared to the control groups [5]. Reviewing all the cases, it has been shown that this increase was due to an accelerated OA process in a few patients, especially those co-treated with NSAIDs. Nevertheless, NGF displays proinflammatory effects, including the stimulation of cytokine and prostaglandin E_2_ (PGE_2_) synthesis, monocyte differentiation, mast cell proliferation and degranulation [6]. Moreover, the injection of NGF into the synovium of rats increased the density of mast cells [7].

Since there is an unmet need for treating pain in OA patients, any explanations on the occurrence of such deleterious effects with anti-NGF drugs are welcome. It has been proposed that pain improvement allows increased joint activity leading to subsequent overuse [8]. More directly, NGF improves ligament healing [9] and decelerates chondrocyte differentiation *in vitro*[10], which is suspected to play an important role in OA progression. In addition, NGF can also display anti-inflammatory effects [11,12]. Notably, NGF inhibited interleukin (IL)-1β-induced tumor necrosis factor-α (TNF-α) production in OA synovial fibroblasts [13].

Inflammatory factors, including IL-1β, TNF-α and adipokines [14] are found in increased levels in OA joints and are proposed to play a role in OA progression, especially by switching chondrocytes towards a catabolic phenotype. IL-1β downregulates the expression of cartilage extracellular matrix components, while it stimulates matrix metalloproteinase (MMP), cytokine and PGE_2_ synthesis. Similarly, adipokines, including visfatin, stimulate the catabolic activity of chondrocytes [14,15]. NGF levels are increased in the synovial fluid of OA patients [16]. NGF and its two receptors, the high-affinity tyrosine kinase A receptor (trkA) and the low-affinity p75 receptor, are expressed by joint cells including chondrocytes [17,18] and are increased in OA cartilage [10,19]. Moreover, NGF synthesis is highly correlated with the degree of OA cartilage degradation in human [19].

Interestingly, NGF expression is induced in an inflammatory context and mediators such as IL-1β and PGE_2_ can stimulate NGF synthesis [13,20]. For example, intra-articular injection of IL-1β induces an increase in NGF levels [21]. However, stimuli responsible for NGF expression and production in OA cartilage remain to be determined. In the present study, we investigated whether mechanical stress and proinflammatory factors, two main determinants of OA, may influence NGF synthesis by chondrocytes. For this purpose, primary cultures of human OA chondrocytes and newborn mouse articular chondrocytes or cartilage explants were stimulated by increasing amounts of IL-1β, PGE_2_, visfatin or by cyclic mechanical compression. NGF mRNA expression and protein released into media were determined.

## Materials and methods

### Primary culture of human chondrocytes

Human cartilage samples were obtained from OA patients undergoing total knee arthroplasty at Saint-Antoine Hospital (Paris, France). Informed consent was obtained for each patient before surgery. The diagnosis of OA was based on clinical and radiographic evaluations according to the criteria of the American College of Rheumatology [22]. Experiments using human samples were approved by the local ethics committee (CPP Ile de France V, 2 May 2012).

Human articular chondrocytes were isolated by enzymatic digestion of cartilage, as previously described [15]. Cells were seeded in 12-well plates (500,000 cells/well) and allowed to grow to confluence in Dulbecco’s modified Eagle’s medium (DMEM) (4.5 mg/L glucose) supplemented with 10% fetal calf serum, 100 IU/mL penicillin, 100 μg/mL streptomycin and 4 mM L-glutamine (Sigma-Aldrich, Saint Quentin Fallavier, France). Cells were then starved in serum-free medium containing 0.3% bovine serum albumin for 24 hours before stimulation.

### Primary culture of mouse chondrocytes

All experiments were performed according to the protocols approved by the French/European ethics committee. Immature murine articular chondrocytes were isolated by enzymatic digestion of articular cartilage from six-day-old newborn animals from one Swiss mouse litter (Janvier Labs, Le Genest Saint Isle, France), according to a previously described procedure [23]. Cells were seeded in 12-well plates (10,000 cells/cm^2^) and allowed to grow for six to seven days in DMEM (1 mg/L glucose) supplemented with 10% fetal calf serum, 100 IU/mL penicillin, 100 μg/mL streptomycin and 4 mM L-glutamine (Sigma-Aldrich). Cells were then starved in serum-free medium containing 0.1% bovine serum albumin for 24 hours before stimulation.

### Cell stimulation

Human OA and murine articular chondrocytes were stimulated with IL-1β (PeproTech, from Tebu Bio, Le Perray-en-Yvelines, France; 0.1 to 10 ng/mL from 2 to 24 hours), PGE_2_ (Cayman Chemical, from SPI-Bio, Montigny-le-Bretonneux, France; 0.1 to 10 μM for 24 hours), or visfatin (Alexis Biochemical, Paris, France; 1 to 10 μg/mL for 24 hours) dissolved in starvation media. In additional experiments, chondrocytes were co-incubated with either IL-1β (1 ng/mL) and indomethacin (Sigma-Aldrich; 0.1 to 10 μM), an inhibitor of the cyclooxygenase activity, or IL-1β (1 ng/mL) and visfatin (5 μg/mL) or visfatin (5 μg/mL) and Apo866 (10 nM) (generously provided by Alexander So; Astellas Pharma, Munich, Germany), an inhibitor of the nicotinamide phosphoribosyltransferase activity of visfatin.

After stimulation, cells were disrupted in lysis buffer (RLT, from Qiagen, Courtaboeuf, France) and conditioned media were stored at -80°C until analysis.

### Transfection of small interfering RNA (siRNA)

Small interfering RNA (siRNA) directed against mouse visfatin was designed and purchased from Ambion Cenix (Austin, TX, USA). The sequence specific for mouse visfatin was forward 5’-GGCACCACUAAUCAUCAGAtt-3’, reverse 5’-UCUGAUGAUUAGUGGUGCCtc-3’.

Mouse chondrocytes were cultured as described above. Confluent cells were removed with trypsin, and 6 × 10^5^ chondrocytes were seeded in 6-cm tissue culture plates and grown for 24 hours, to 70 to 80% confluence. Normal growth medium containing 10% fetal bovine serum was changed prior to siRNA transfection. Transfections were performed as described for the RNAi Starter Kit (Qiagen). Cells were incubated for 18 hours with siRNA and transfection reagent, rinsed twice with phosphate-buffered saline (PBS), and placed in DMEM (1 mg/L glucose) supplemented with penicillin, streptomycin, and L-glutamine containing 1% BSA, with or without IL-1β (10 ng/ml) for 24 hours. Transfection of siRNA against MAPK-1, a ubiquitously produced mouse cell protein, was used as a positive control. A nonsilencing siRNA that has no homology with any known mammalian gene (RNAi Starter Kit) and scrambled siRNA (Ambion) were used as negative controls.

### Mechanical compression

Mechanical compression was applied on costal cartilage, as previously described [24]. Briefly, ribs cages from six-day-old newborns from one Swiss mouse litter (Janvier Labs) were harvested and cartilage was separated from bone and soft tissues. Immediately after dissection, each sample, consisting of 50 mg of cartilage explants, were placed into Biopress culture plates (Flexercell International, Hillsborough, NC, USA) in DMEM (1 mg/L glucose) supplemented with 30 mM Hepes, 100 IU/mL penicillin, 100 μg/mL streptomycin and 4 mM L-glutamine (Sigma-Aldrich). During 4 to 24 hours, intermittent compression was applied by the Biopress system (Flexercell International) using a sinusoidal waveform at 0.5 Hz and 1.0 MPa of magnitude (Figure S1 in Additional file 1). Control explants were kept in unloaded condition.

After compression, cartilage explants frizzed in liquid nitrogen and conditioned media were stored at -80°C until analysis.

### RNA extraction, reverse transcription and quantitative real-time PCR

Total RNA was extracted from cultured cells with RNeasy Mini kit (Qiagen) according to the manufacturer’s instructions. Total RNA was reverse transcribed with Omniscript RT kit according to the manufacturer's instructions (Qiagen). NGF mRNA expression was analyzed by quantitative real-time PCR using the Light Cycler 480 (Roche Diagnostics, Meylan, France). The equivalent of 5 to 10 ng initial RNA was subjected to PCR amplification in a 12 μl final volume using specific primers at 10 μM and LC 480 SYBR Green I Master kit (Roche Diagnostics). PCR amplification conditions were: initial denaturation for 5 min at 95°C followed by 40 cycles consisting of 10 s at 95°C, 15 s at 60°C and 10 s at 72°C. Product formation was detected at 72°C in the fluorescein isothiocyanate channel. The generation of specific PCR products was confirmed by melting curve analysis. For each PCR, cDNAs were run in duplicate in parallel with serial dilutions of a cDNA mixture tested for each primer pair to generate a standard linear curve, which was used to estimate the amplification efficiency for NGF and HPRT. The relative mRNA expression of NGF normalized to that of HPRT, used as internal reference gene, was determined by the Efficiency method of the LightCycler 480 Software.

The specific oligonucleotide primers used were as follows: human NGF, sense ^5’^GTCATCATCCCATCCCATCT^3’^ and anti-sense ^5’^AGTACTGTTTGAATACACTGTTGTTAAT^3’^; mouse NGF, sense ^5’^CACCCACCCAGTCTTCC^3’^ and anti-sense ^5’^CTCGGCACTTGGTCTCAAA^3’^; human GAPDH, sense ^5’^CCATCACCATCTTCCA^3’^ and anti-sense ^5’^CCTTCTCCATGGTGGT^3’^; mouse HPRT, sense, ^5’^GCTGGTGAAAAGGACCTCT^3’^ and anti-sense, ^5’^CACAGGACTAGAACACCTGC^3’^.

### NGF assay

NGF concentrations in the medium were measured using an enzyme-linked immunosorbent assay (ELISA) kit from Promega (Charbonnières, France) according to the manufacturer’s instructions. The detection limit of NGF was 7.8 pg/mL and the intra-assay coefficients of variance was 4.2%. The NGF concentrations were analyzed in duplicate at serial dilution and were read against a standard curve.

### Statistical analysis

Results are expressed as means ± standard deviation (SD). Results were compared using one-way factorial analysis (analysis of variance) followed by the Scheff´s *F* test (Statview software, version 4.57; Abacus Concepts Inc., Berkeley, CA, USA). Statistical significance was accepted for *P <*0*.*05.

## Results

### Induction of NGF expression and release by costal cartilage explants in response to mechanical stress

**Figure 1:**
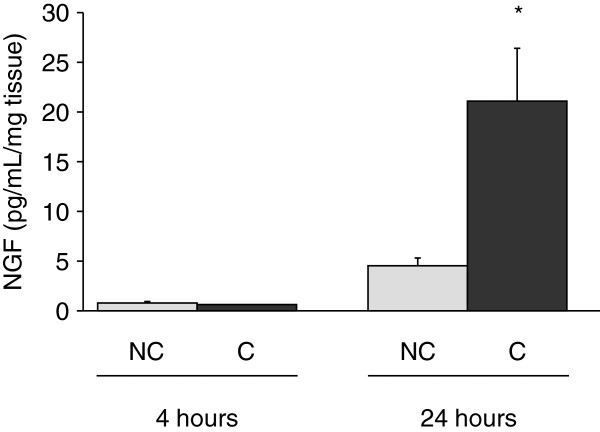
**Stimulation of NGF release by cartilage compression.** Explants of costal cartilages from Swiss mice were compressed (C) or not (NC) for 4 and 24 hours (n = 4). NGF protein levels were measured in conditioned media. *P <0.05. NGF, nerve growth factor.

Since mechanical overload is considered as a strong inducer of cartilage damage and pain, the influence of *in vitro* mechanical compression on NGF release by costal cartilage explants was investigated. For this purpose, murine costal cartilages were subjected to intermittent compression for 4 and 24 hours. Costal cartilage explants constitutively released low levels of NGF that accumulated within conditioned media between 4 and 24 hours (Figure 1A). Compression for 4 hours did not stimulate NGF release. In contrast, accumulation of NGF into tissue-conditioned media was significantly increased after compression for 24 hours (4.7-fold increase versus control, *P* = 0.04) (Figure 1).

### Induction of NGF expression and release by articular chondrocytes in response to visfatin

**Figure 2:**
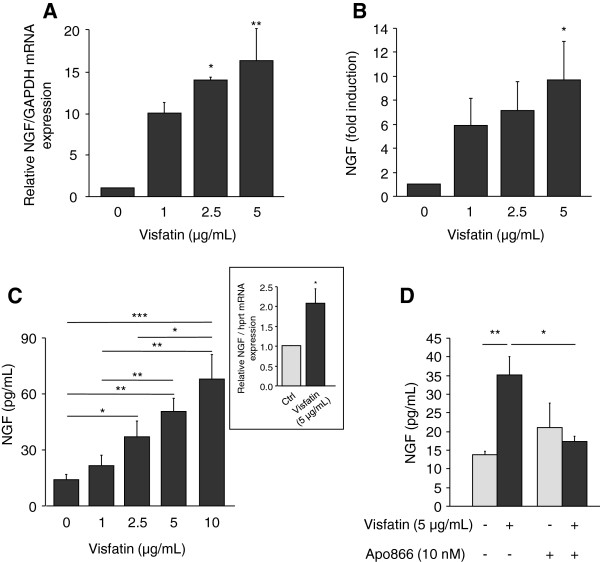
**Visfatin induces NGF expression and release by chondrocytes.** Human OA chondrocytes were stimulated for 24 hours with increasing concentrations of visfatin (0, 1, 2.5 and 5 μg/mL) and NGF mRNA expression **(A)** and release into cell supernatant **(B)** were measured by quantitative RT-PCR and ELISA, respectively (n = 3) Control cells released 20.4 ± 12.2 pg/mL NGF. **(C)** Primary cultures of mouse articular chondrocytes were stimulated for 24 hours with increasing concentration of visfatin (0, 1, 2.5, 5 and 10 μg/mL) and NGF levels were measured in conditioned media (n = 3). Inset: NGF mRNA expression was determined by quantitative RT-PCR in control cells and chondrocytes stimulated with visfatin (5 μg/mL) for 24 hours. The amount of NGF mRNA was normalized against the amount of HPRT mRNA measured in the same cDNA. **(D)** Mouse articular chondrocytes were incubated with or without Apo866 (10 nM) for 4 hours and then treated or not with visfatin (5 μg/mL) for 24 hours (n = 3). NGF release was determined into cell-conditioned media. *P <0.05, **P <0.01, ***P <0.001. ELISA, enzyme-linked immunosorbent assay; NGF, nerve growth factor; OA, osteoarthritis.

Visfatin is an adipokine found in OA synovial fluids [25], which displays catabolic activity on chondrocytes [15]. We thus determined whether NGF expression and release could be modulated by visfatin. Human OA and mouse articular chondrocytes were stimulated with increasing concentrations of visfatin (1, 2.5, 5 and 10 μg/mL) for 24 hours and NGF mRNA expression and release into conditioned media was measured. Both human and mouse articular chondrocytes spontaneously synthesized NGF mRNA and protein (Figure 2). Visfatin induced a dose-dependent increase in NGF mRNA expression in human OA chondrocytes (Figure 2A). The stimulation of NGF mRNA expression was observed with 1 μg/mL visfatin and became statistically significant with 2.5 μg/mL visfatin (14-fold increase as compared to control, *P* = 0.02). Consequently, an accumulation of NGF into human chondrocyte-conditioned media was measured after visfatin stimulation (Figure 2B). Similarly, a dose-dependent increased accumulation of NGF was observed in conditioned media of mouse articular chondrocytes (Figure 2C). A significant increase, as compared to unstimulated chondrocytes, was obtained with 2.5 μg/mL visfatin (2.6-fold versus control, *P* = 0.03) and the highest concentration of visfatin (10 μg/mL) induced the highest accumulation of NGF into cell supernatant (4.9-fold versus control, *P* = 0.0003) (Figure 2C). Visfatin-mediated release of NGF was associated with an increase in NGF mRNA expression (2-fold versus control, *P* = 0.045) (Figure 2C, inset).

In addition to its cytokine-like activity, visfatin displays a nicotinamide phosphoribosyltransferase (NAMPT) enzymatic activity responsible for the synthesis of nicotinamide adenine dinucleotide (NAD). Several lines of evidence support the involvement of this enzymatic activity in the cellular effects of visfatin/NAMPT [26]. To investigate the involvement of visfatin/NAMPT enzymatic activity in NGF stimulation, chondrocytes were pretreated with 10 nM Apo866, a specific inhibitor of NAMPT [27], for 4 hours before the addition of exogenous visfatin/NAMPT (5 μg/mL). Apo866 treatment led to a 56% and 68% drop in NAD concentration in control and visfatin/NAMPT-treated chondrocytes (*P* = 0.0003 and *P* <0.0001, respectively) (not shown). The inhibition of visfatin/NAMPT enzymatic activity by Apo866 prevented the increase in NGF synthesis (*P* = 0.02 versus visfatin/NAMPT-treated cells without Apo866) (Figure 2D). In contrast, no modification of spontaneous release of NGF by chondrocytes was observed in the presence of Apo866.

### Induction of NGF expression and release by articular chondrocytes in response to IL-1β

**Figure 3:**
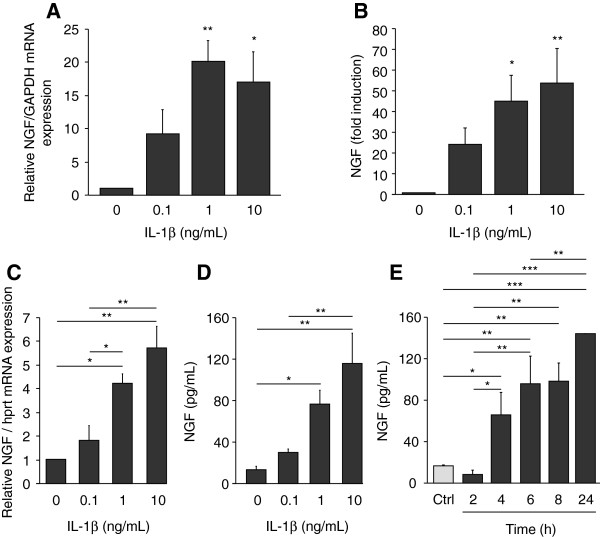
**Stimulation of articular chondrocyte expression and release of NGF by IL-1β.** Human OA chondrocytes were stimulated for 24 hours with increasing concentrations of IL-1β (0, 0.1, 1 and 10 ng/mL) and NGF mRNA expression **(A)** and release into cell supernatant **(B)** were measured by quantitative RT-PCR and ELISA, respectively (n = 4). Control cells released 19.2 ± 8.7 pg/mL NGF. **(C-E)** Primary cultures of mouse articular chondrocytes were stimulated either with increasing concentrations of IL-1β (0, 0.1, 1 and 10 ng/mL) for 24 hours (n = 3) **(C and D)** or with IL-1β (10 ng/mL) for 2, 4, 6, 8 and 24 hours (n = 3) **(E)**. As a control (ctrl), chondrocytes were incubated without IL-1β for 24 hours **(E)**. NGF mRNA expression was determined by quantitative RT-PCR **(C)**. The amount of NGF mRNA was normalized against the amount of HPRT mRNA measured in the same cDNA. NGF protein levels were measured in conditioned media by ELISA **(D and E)**. *P <0.05, **P <0.01, ***P <0.001. ELISA, enzyme-linked immunosorbent assay; IL-1β, interleukin 1β; NGF, nerve growth factor; OA, osteoarthritis.

We next investigated the action of IL-1β on NGF mRNA expression and release by human OA and murine articular chondrocytes. IL-1β dose-dependently increased NGF mRNA expression in human OA articular chondrocytes (Figure 3A). A 9.3-fold induction was observed with 0.1 ng/mL IL-1β and became statistically significant with 1 ng/mL (*P* = 0.004). This was associated with increased levels of NGF measured in conditioned media of chondrocytes stimulated with IL-1β (Figure 3B). IL-1β also stimulated NGF mRNA expression in mouse articular chondrocytes since a 1.8-fold induction was observed with 0.1 ng/mL IL-1β and became statistically significant with 1 ng/mL (*P* = 0.01). NGF mRNA expression further increased using 10 ng/mL of IL-1β (5.7-fold compared to control, *P* = 0.004). IL-1β-induced NGF mRNA expression paralleled the release of NGF protein into cell-conditioned media (Figure 3C and D). NGF protein release was slightly increased at 0.1 ng/mL and this increase became significant at 1 ng/mL IL-1β (*P* = 0.024). In the time-course experiment, IL-1β-induced NGF release was observed after a 4-hour stimulation (4.2-fold increase versus control, *P* = 0.049) and further increased up to 24 hours (9.2-fold increase versus control, *P* = 0.0005) (Figure 3E).

**Figure 4:**
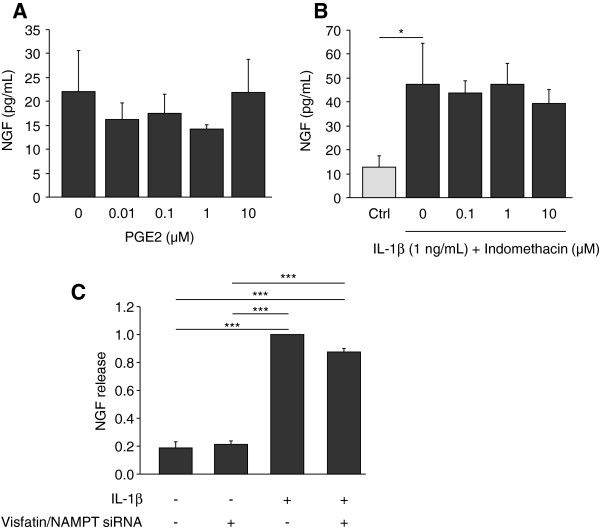
**PGE**_**2 **_**and endogenous visaftin/NAMPT are not involved in IL-1β-mediated stimulation of NGF release by articular chondrocytes. (A and B)** Primary cultures of mouse articular chondrocytes were stimulated for 24 hours either with increasing concentrations of PGE_2_ (0, 0.01, 0.01, 1 and 10 ng/mL) (n = 3) **(A)** or with IL-1β (1 ng/mL) in the presence of increasing concentrations of indomethacin (0, 0.1, 1, 10 μM) (n = 3) **(B)**. As a control (ctrl), chondrocytes were incubated without IL-1β for 24 hours **(B)**. NGF protein levels were measured in conditioned media by ELISA. **(C)** Primary culture of mouse articular chondrocytes were transfected by siRNA targeting visfatin/NAMPT before stimulation with IL-1β (10 ng/mL) for 24 hours (n = 2). Each experiment was performed in triplicate. Results are expressed as NGF release as compared to IL-1β-stimulated chondrocytes. *P <0.05, ***P <0.001. ELISA, enzyme-linked immunosorbent assay; IL-1β, interleukin 1β; NAMPT, nicotinamide phosphoribosyltransferase activity. Supress activity; NGF, nerve growth factor; PGE_2_, prostaglandin E_2_; siRNA, small interfering RNA.

IL-1β is a strong inducer of PGE_2_ synthesis by cartilage and chondrocytes. However, PGE_2_ was not involved in IL-1β-mediated NGF stimulation. Indeed, PGE_2_ (0.01 to 10 μM) did not stimulate NGF release by articular chondrocytes (Figure 4A). Furthermore, indomethacin (0.1 to 10 μM), a specific inhibitor of cyclooxygenase enzymatic activity, was unable to prevent the release of NGF induced by IL-1β (1 ng/mL) (Figure 4B), although it efficiently inhibited the IL-1β-mediated production of PGE_2_ by chondrocytes (not shown).

Since some of the effects of IL-1β on chondrocytes involve visfatin/NAMPT [15], consequences of visfatin/NAMPT knockdown on IL-1β-mediated NGF stimulation were assessed (Figure 4C). No effect of visfatin/NAMPT siRNA on the constitutive release of NGF was observed. Similarly, the potent stimulation of NGF secretion induced by IL-1β (10 ng/mL) (5-fold as compared to control, *P* >0.0002) was not blocked by visfatin/NAMPT siRNA, suggesting that endogenous visfatin/NAMPT is not involved in the IL-1β-stimulated release of NGF by chondrocytes (Figure 4C).

**Figure 5:**
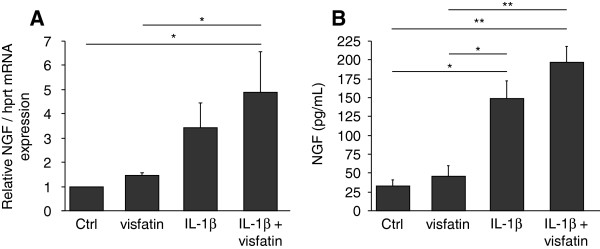
**Potentiation of IL-1β-stimulated NGF expression and release by visfatin/NAMPT.** Primary cultures of mouse articular chondrocytes were stimulated or not for 24 hours either with IL-1β (1 ng/mL) or visfatin/NAMPT (5 μg/mL) or with IL-1β (1 ng/mL) and visfatin/NAMPT (5 μg/mL) (n = 3). The mRNA expression of NGF **(A)** and the NGF protein levels in chondrocyte conditioned media were determined **(B)**. *P <0.05, ***P <0.001. IL-1β, interleukin 1β; NAMPT, nicotinamide phosphoribosyltransferase activity. Supress activity; NGF, nerve growth factor.

### Potentiation of IL-1β-stimulated NGF expression and release by visfatin/NAMPT

To determine whether the stimulation of NGF mRNA expression and release by IL-1β and visfatin/NAMPT could be additive, murine articular chondrocytes were co-stimulated with IL-1β (1 ng/mL) and visfatin/NAMPT (5 μg/mL). A slight potentiation of IL-1β-mediated stimulation of NGF was observed both at mRNA (1.4-fold) and protein levels (1.3-fold) when chondrocytes were co-stimulated with visfatin/NAMPT (Figure 5). This potentiation was similar to the stimulatory effect of visfatin/NAPMT since visfatin/NAMPT induced a 1.5-fold and 1.4-fold stimulation of NGF mRNA expression and release, respectively (Figure 5). Compression did not further increase the release of NGF from cartilage induced by either IL-1β or visfatin/NAMPT or IL-1β and visfatin/NAMPT (not shown).

## Discussion

Several lines of evidence highlight NGF as an attractive therapeutic target to control OA pain. NGF is a key factor in inflammation-associated hyperalgesia, whose protein is detected in OA synovial fluid [16] and mRNA expression is enhanced in OA chondrocytes [19]. NGF may also mediate joint damage [6]. Preclinical studies on experimental models of joint pain [1,3] and clinical trials on OA patients [2,4] thus evidenced the efficiency of NGF targeting in the reduction of joint pain. However, the increased number of total joint prosthesis due to acceleration in lesion progression in some patients decided the FDA to stop all clinical trials. A better understanding of the involvement of NGF in OA is therefore required. In this study, we investigated whether proinflammatory factors and mechanical compression, the main determinants of OA, may lead to the expression and the release of NGF by chondrocytes.

We first show that human OA chondrocytes as well as mouse cartilage and articular chondrocytes constitutively expressed NGF mRNA and released NGF protein into conditioned media, as already reported by Iannone *et al.*[19] in human normal articular chondrocytes. We also observed an increase in NGF expression and release in response to OA stimuli, consistently with the enhanced expression of NGF by OA chondrocytes [19]. In this context, we show for the first time that dynamic compression induced the release of NGF by cartilage, highlighting NGF as a mechanosensitive gene in chondrocytes. This result is in accordance with data showing that mechanical stretch can modulate NGF expression with an induction observed in smooth muscle cells [28] and sympathetic neurocytes [29], whereas a downregulation was obtained in rat cardiomyocytes [30].

We also show that IL-1β, which is considered as the main inflammatory mediator involved in cartilage degradation in OA, induced in a time- and dose-dependent manner an increase in NGF expression and release by both human and mouse articular chondrocytes. Similarly, IL-1β has been reported to stimulate the expression of NGF in several cell types, including human nucleus pulposus cells isolated from patients with intervertebral disc (IVD) degeneration [31-33]. The intra-articular injection of IL-1β induced an increase in NGF levels [21] and a positive correlation between IL-1β and NGF immunostaining in human IVD tissues was observed [34].

In addition to its well-known pro-degradative property on cartilage, IL-1β plays also a role in joint pain. Indeed, IL-1β levels in OA synovial fluids are associated with pain and hyperalgesia of the temporomandibular joint [35] and the synovial mRNA expression of IL-1β is correlated with the degree of pain in rotator cuff diseases [36]. OA patients showing a 2-fold higher expression of IL-1β by peripheral blood leukocytes compared to non-OA controls have higher pain scores and risks of radiographic OA progression than patients displaying similar peripheral blood leukocyte IL-1β expression than non-OA controls [37]. Furthermore, a reduced pain was obtained following the treatment of patients with chronic active gouty arthritis with rilonacept, an inhibitor of IL-1β [38]. Similarly, the subcutaneous injection of canakinumab, an anti-human IL-1β antibody, to patients with cryopyrin-associated periodic syndrome provoked a sustained remission of symptoms, including pain [39]. All these data suggest that IL-1β-mediated stimulation of NGF expression by chondrocytes may account for the pain associated with IL-1β levels in OA joints.

Adipokines have recently been proposed as molecular mediators probably involved in OA [40]. Among them, visfatin/NAMPT was characterized as a visceral fat cytokine, a 52 kDa protein with insulin mimetic activity [41]. Visfatin/NAMPT is released by all joint tissues in OA patients [25] and its concentration in synovial fluids is linked to cartilage degradation biomarkers [42]. Visfatin/NAMPT stimulates chondrocyte catabolic activity measured by PGE_2_ and protease synthesis [15] and induces the expression of several proinflammatory cytokines by chondrocytes (Laiguillon, submitted). Our results show that visfatin also stimulated both NGF expression and release by human OA and mouse articular chondrocytes, suggesting that it may favor OA pain in addition to mediate cartilage damage.

In addition to its cytokine-like activity, visfatin displays a NAMPT enzymatic activity responsible for the synthesis of NAD. NAD biosynthetic activity of visfatin/NAMPT is involved in some cellular effects of extracellular visfatin/NAMPT [43-45]. Interestingly, the pharmacological inhibition of visfatin/NAMPT by Apo866, a specific inhibitor of NAMPT activity, prevents arthritis progression [46,47]. In a previous study, we showed that blocking NAMPT enzymatic activity inhibited the catabolic response of chondrocytes to exogenous visfatin/NAMPT [26]. Similarly, we show here that NAMPT enzymatic activity was also crucial for the visfatin/NAMPT-mediated NGF stimulation in chondrocytes, suggesting the involvement of NAD in the signaling pathway of visfatin/NAMPT leading to NGF expression. NAD-consuming proteins, including sirtuins and poly(ADP-ribose) polymerases (PARPs), are known actors in the expression of many genes [48]. However, nothing is known about a putative role of such proteins in the expression of NGF. In addition, NAD consumption generates second messengers, which contribute to intracellular calcium signaling [49]. Intracellular calcium can regulate the expression of NGF [28,30].

IL-1β-mediated NGF expression involves nuclear factor kappa B (NF-κB) activity [50]. Interestingly, the proinflammatory effect of visfatin/NAMPT requires NF-κB activity in a NAMPT enzymatic activity-dependent manner. Some of the effects of IL-1β involve endogenous visfatin/NAMPT, as demonstrated by siRNA strategy [15]. However, we did not obtain any inhibition of IL-1β-mediated NGF secretion by using siRNA targeting endogenous visfatin/NAMPT, showing that endogenous visfatin/NAMPT is not involved in the stimulation of NGF induced by IL-1β. As NAD-derived second messengers activate intracellular calcium signaling, these results could explain why intracellular calcium signaling is not involved in IL-1β-mediated NGF expression [51]. Taken together, our results support that different signaling pathways, requiring endogenous visfatin/NAMPT or not, can be activated by IL-1β. Our results also show that the stimulation of NGF expression and release in response to IL-1β and visfatin/NAMPT involves different pathways, thus explaining the potentiation of IL-1β-mediated stimulation of NGF expression and release by visfatin/NAMPT.

Interestingly, mechanical loading, IL-1β and visfatin all induce PGE_2_ production by cartilage and chondrocytes [15,52,53]. PGE_2_ was reported to induce the expression of NGF in mouse astrocyte cultures [54,55]. Blocking the generation of prostaglandins by celecoxib (Celebrex) prevented the increased secretion of NGF by tooth perfusates in rats after an inflammatory stress [56]. PGE_2_ may be also involved in IL-1β-mediated pain [57]. PGE_2_ may thus represent a common way for NGF expression in chondrocytes in response to OA stimuli. However, our results show that PGE_2_, whatever the concentration used, was unable to induce NGF expression and release in chondrocytes. In addition, indomethacin, an inhibitor of cyclooxygenase enzymatic activity, did not prevent the IL-1β-mediated increase in NGF expression in chondrocytes. These results suggest that the increased chondrocyte expression of NGF in response to several OA stimuli did not involve PGE_2_. In contrast, other studies showed that PGE_2_ modulates the expression and synthesis of NGF. Friedman *et al.* reported that indomethacin partly blocked the increase in NGF production in response to IL-1β in embryonic rat hippocampal cultures [20]. PGE_2_ stimulates the secretion of NGF in astrocytes [54,55], whereas in 3T3-L1 adipocytes it decreases both NGF expression and synthesis [58]. Thus, the influence of PGE_2_ on NGF synthesis appears to be cell-type-dependent and may be related to the differential expression pattern of PGE_2_ receptor in these cells. High amounts of E-type prostanoid (EP)3 receptor are indeed expressed in mature adipocytes [59], whereas chondrocytes express low levels of EP3 [60,61].

## Conclusions

OA is a painful disease, in which NGF plays a crucial role. Indeed, pivotal clinical trials have demonstrated that counteracting NGF leads to a dramatic reduction of OA pain [2]. Unfortunately, all the NGF antibodies in development have induced an acceleration of the OA process in a subgroup of patients. It is thus mandatory to accumulate more information on the mechanism of action of NGF in order to enhance the benefit-risk ratio of this new family of effective symptomatic drugs. Our results show that two main OA determinants, mechanical stress and proinflammatory factors, including IL-1β and extracellular visfatin/NAMPT, stimulated the expression and the release of NGF by articular chondrocytes. These results suggest that OA pain may involve the release of NGF by chondrocytes in response to the increased levels of IL-1β and extracellular visfatin/NAMPT, and to the increased mechanical loading of cartilage.

## Abbreviations

DMEM: Dulbecco’s modified Eagle’s medium; ELISA: enzyme-linked immunosorbent assay; IL-1β: interleukin 1β; IVD: intervetebral disc; MMP: matrix metalloproteinase; NAMPT: nicotinamide phosphoribosyltransferase activity. Supress activity; NGF: nerve growth factor; NSAID: non-steroidal anti-inflammatory drug; OA: osteoarthritis; PGE2: prostaglandin E_2_; siRNA: small interfering RNA; TNF-α: tumor necrosis factor-α; trkA: tyrosine kinase A receptor.

## Competing interests

All authors state that there is no conflict of interests.

## Author’s contributions

EP participated in the conception and design of the study, data collection and analysis, the manuscript writing and final approval of the manuscript. SP carried out data collection and analysis, and participated in the critical revision and final approval of the manuscript. MG carried out data collection and analysis, and participated in the critical revision and final approval of the manuscript. AP contributed to data collection and analysis and the final approval of the manuscript. LS contributed to data collection and analysis and the final approval of the manuscript. MCL contributed to data collection and analysis and the final approval of the manuscript. FB participated in the conception and design of the study, provided financial support, and contributed to manuscript writing and the final approval of the manuscript. XH participated in the conception and design of the study, data collection and analysis, the manuscript writing, and final approval of the manuscript. All authors read and approved the final manuscript.

## Supplementary Material

Additional file 1: Figure S1 Compression system**. (A)** FX-4000C^™^ Flexercell^™^ Compression Plus^™^ System (Flexcell International Corp., Hillsborough, NC, USA). A positive pressure compresses samples between a piston and stationary platen on the BioPress^™^ culture. **(B)** Schematic diagram of the BioPress^™^ culture plate compression chamber in uncompressed or compressed position. Click here for file

## References

[B1] Iwakura N Ohtori S Orita S Yamashita M Takahashi K Kuniyoshi K Role of low-affinity nerve growth factor receptor inhibitory antibody in reducing pain behavior and calcitonin gene-related Peptide expression in a rat model of wrist joint inflammatory pain J Hand Surg Am 2010 16 267 273 10.1016/j.jhsa.2009.10.030 20060234

[B2] Lane NE Schnitzer TJ Birbara CA Mokhtarani M Shelton DL Smith MD Brown MT Tanezumab for the treatment of pain from osteoarthritis of the knee N Engl J Med 2010 16 1521 1531 10.1056/NEJMoa0901510 20942668 PMC6896791

[B3] McNamee KE Burleigh A Gompels LL Feldmann M Allen SJ Williams RO Dawbarn D Vincent TL Inglis JJ Treatment of murine osteoarthritis with TrkAd5 reveals a pivotal role for nerve growth factor in non-inflammatory joint pain Pain 2010 16 386 392 10.1016/j.pain.2010.03.002 20350782

[B4] Brown MT Murphy FT Radin DM Davignon I Smith MD West CR Tanezumab reduces osteoarthritic hip pain: results of a randomized, double-blind, placebo-controlled phase 3 trial Arthritis Rheum 2013 16 1795 1803 23553790 10.1002/art.37950

[B5] Hochberg MC Abramson SB Hungerford DS EM Vignon EP Smith MC Tive L Verburg KM West CR Adjudication of reported serious adverse joint events in the Tanezumab clinical development program Arthritis Rheum 2012 16 S113 10.1002/art.39492 26554876

[B6] Seidel MF Herguijuela M Forkert R Otten U Nerve growth factor in rheumatic diseases Semin Arthritis Rheum 2010 16 109 126 10.1016/j.semarthrit.2009.03.002 19481238

[B7] Aloe L Tuveri MA Levi-Montalcini R Nerve growth factor and distribution of mast cells in the synovium of adult rats Clin Exp Rheumatol 1992 16 203 204 1505118

[B8] Smelter E Hochberg MC New treatments for osteoarthritis Curr Opin Rheumatol 2013 16 310 316 10.1097/BOR.0b013e32835f69b4 23425965

[B9] Mammoto T Seerattan RA Paulson KD Leonard CA Bray RC Salo PT Nerve growth factor improves ligament healing J Orthop Res 2008 16 957 964 10.1002/jor.20615 18302239

[B10] Huang H Shank G Ma L Tallents R Kyrkanides S Nerve growth factor induced after temporomandibular joint inflammation decelerates chondrocyte differentiation Oral Dis 2013 16 604 610 10.1111/odi.12045 23231346

[B11] Amico-Roxas M Caruso A Leone MG Scifo R Vanella A Scapagnini U Nerve growth factor inhibits some acute experimental inflammations Arch Int Pharmacodyn Ther 1989 16 269 285 2774768

[B12] Nordell VL Lewis DK Bake S Sohrabji F The neurotrophin receptor p75NTR mediates early anti-inflammatory effects of estrogen in the forebrain of young adult rats BMC Neurosci 2005 16 58 10.1186/1471-2202-6-58 16156894 PMC1239918

[B13] Manni L Lundeberg T Fiorito S Bonini S Vigneti E Aloe L Nerve growth factor release by human synovial fibroblasts prior to and following exposure to tumor necrosis factor-alpha, interleukin-1 beta and cholecystokinin-8: the possible role of NGF in the inflammatory response Clin Exp Rheumatol 2003 16 617 624 14611111

[B14] Gomez R Conde J Scotece M Gomez-Reino JJ Lago F Gualillo O What's new in our understanding of the role of adipokines in rheumatic diseases? Nat Rev Rheumatol 2011 16 528 536 10.1038/nrrheum.2011.107 21808287

[B15] Gosset M Berenbaum F Salvat C Sautet A Pigenet A Tahiri K Jacques C Crucial role of visfatin/pre-B cell colony-enhancing factor in matrix degradation and prostaglandin E2 synthesis in chondrocytes: possible influence on osteoarthritis Arthritis Rheum 2008 16 1399 1409 10.1002/art.23431 18438860

[B16] Aloe L Tuveri MA Carcassi U Levi-Montalcini R Nerve growth factor in the synovial fluid of patients with chronic arthritis Arthritis Rheum 1992 16 351 355 10.1002/art.1780350315 1536673

[B17] Gigante A Bevilacqua C Pagnotta A Manzotti S Toesca A Greco F Expression of NGF, Trka and p75 in human cartilage Eur J Histochem 2003 16 339 344 14706929

[B18] Grimsholm O Guo Y Ny T Forsgren S Expression patterns of neurotrophins and neurotrophin receptors in articular chondrocytes and inflammatory infiltrates in knee joint arthritis Cells Tissues Organs 2008 16 299 309 10.1159/000121432 18349525

[B19] Iannone F De Bari C Dell'Accio F Covelli M Patella V Lo Bianco G Lapadula G Increased expression of nerve growth factor (NGF) and high affinity NGF receptor (p140 TrkA) in human osteoarthritic chondrocytes Rheumatology (Oxford) 2002 16 1413 1418 10.1093/rheumatology/41.12.1413 12468822

[B20] Friedman WJ Larkfors L Ayer-LeLievre C Ebendal T Olson L Persson H Regulation of beta-nerve growth factor expression by inflammatory mediators in hippocampal cultures J Neurosci Res 1990 16 374 382 10.1002/jnr.490270316 2129046

[B21] Manni L Aloe L Role of IL-1 beta and TNF-alpha in the regulation of NGF in experimentally induced arthritis in mice Rheumatol Int 1998 16 97 102 10.1007/s002960050065 9833249

[B22] Altman R Asch E Bloch D Bole G Borenstein D Brandt K Christy W Cooke TD Greenwald R Hochberg M Howell D Kaplan D Koopman W Longley S III Mankin H McShane DJ Medsger T Jr Meenan R Mikkelsen W Moskowitz R Murphy W Rotschild B Segal M Sokoloff L Wolfe F Development of criteria for the classification and reporting of osteoarthritis, Classification of osteoarthritis of the knee. Diagnostic and Therapeutic Criteria Committee of the American Rheumatism Association Arthritis Rheum 1986 16 1039 1049 10.1002/art.1780290816 3741515

[B23] Salvat C Pigenet A Humbert L Berenbaum F Thirion S Immature murine articular chondrocytes in primary culture: a new tool for investigating cartilage Osteoarthritis Cartilage 2005 16 243 249 10.1016/j.joca.2004.11.008 15727891

[B24] Gosset M Berenbaum F Levy A Pigenet A Thirion S Saffar JL Jacques C Prostaglandin E2 synthesis in cartilage explants under compression: mPGES-1 is a mechanosensitive gene Arthritis Res Ther 2006 16 R135 10.1186/ar2024 16872525 PMC1779392

[B25] Chen WP Bao JP Feng J Hu PF Shi ZL Wu LD Increased serum concentrations of visfatin and its production by different joint tissues in patients with osteoarthritis Clin Chem Lab Med 2010 16 1141 1145 20482384 10.1515/CCLM.2010.230

[B26] Jacques C Holzenberger M Mladenovic Z Salvat C Pecchi E Berenbaum F Gosset M Proinflammatory actions of visfatin/nicotinamide phosphoribosyltransferase (Nampt) involve regulation of insulin signaling pathway and Nampt enzymatic activity J Biol Chem 2012 16 15100 15108 10.1074/jbc.M112.350215 22399297 PMC3340217

[B27] Hasmann M Schemainda I FK866, a highly specific noncompetitive inhibitor of nicotinamide phosphoribosyltransferase, represents a novel mechanism for induction of tumor cell apoptosis Cancer Res 2003 16 7436 7442 14612543

[B28] Clemow DB Steers WD Tuttle JB Stretch-activated signaling of nerve growth factor secretion in bladder and vascular smooth muscle cells from hypertensive and hyperactive rats J Cell Physiol 2000 16 289 300 10.1002/(SICI)1097-4652(200006)183:3<289::AID-JCP1>3.0.CO;2-6 10797303

[B29] Rana OR Schauerte P Hommes D Schwinger RH Schroder JW Hoffmann R Saygili E Mechanical stretch induces nerve sprouting in rat sympathetic neurocytes Auton Neurosci 2010 16 25 32 10.1016/j.autneu.2010.01.003 20122881

[B30] Rana OR Saygili E Meyer C Gemein C Kruttgen A Andrzejewski MG Ludwig A Schotten U Schwinger RH Weber C Weis J Mischke K Rassaf T Kelm M Schauerte P Regulation of nerve growth factor in the heart: the role of the calcineurin-NFAT pathway J Mol Cell Cardiol 2009 16 568 578 10.1016/j.yjmcc.2008.12.006 19150448

[B31] Abe Y Akeda K An HS Aoki Y Pichika R Muehleman C Kimura T Masuda K Proinflammatory cytokines stimulate the expression of nerve growth factor by human intervertebral disc cells Spine (Phila Pa 1976) 2007 16 635 642 10.1097/01.brs.0000257556.90850.53 17413467

[B32] Purmessur D Freemont AJ Hoyland JA Expression and regulation of neurotrophins in the nondegenerate and degenerate human intervertebral disc Arthritis Res Ther 2008 16 R99 10.1186/ar2487 18727839 PMC2575613

[B33] Taishi P Churchill L De A Obal F Jr Krueger JM Cytokine mRNA induction by interleukin-1beta or tumor necrosis factor alpha in vitro and in vivo Brain Res 2008 16 89 98 18620339 10.1016/j.brainres.2008.05.067 PMC2642478

[B34] Lee JM Song JY Baek M Jung HY Kang H Han IB Kwon YD Shin DE Interleukin-1beta induces angiogenesis and innervation in human intervertebral disc degeneration J Orthop Res 2011 16 265 269 10.1002/jor.21210 20690185

[B35] Alstergren P Ernberg M Kvarnstrom M Kopp S Interleukin-1beta in synovial fluid from the arthritic temporomandibular joint and its relation to pain, mobility, and anterior open bite J Oral Maxillofac Surg 1998 16 1059 1065 discussion 1066 10.1016/S0278-2391(98)90256-7 9734768

[B36] Gotoh M Hamada K Yamakawa H Yanagisawa K Nakamura M Yamazaki H Ueyama Y Tamaoki N Inoue A Fukuda H Interleukin-1-induced subacromial synovitis and shoulder pain in rotator cuff diseases Rheumatology (Oxford) 2001 16 995 1001 10.1093/rheumatology/40.9.995 11561109

[B37] Attur M Belitskaya-Levy I Oh C Krasnokutsky S Greenberg J Samuels J Smiles S Lee S Patel J Al-Mussawir H McDaniel G Kraus VB Abramson SB Increased interleukin-1beta gene expression in peripheral blood leukocytes is associated with increased pain and predicts risk for progression of symptomatic knee osteoarthritis Arthritis Rheum 2011 16 1908 1917 10.1002/art.30360 21717421 PMC3128429

[B38] Terkeltaub R Sundy JS Schumacher HR Murphy F Bookbinder S Biedermann S Wu R Mellis S Radin A The interleukin 1 inhibitor rilonacept in treatment of chronic gouty arthritis: results of a placebo-controlled, monosequence crossover, non-randomised, single-blind pilot study Ann Rheum Dis 2009 16 1613 1617 10.1136/ard.2009.108936 19635719 PMC2732898

[B39] Kone-Paut I Lachmann HJ Kuemmerle-Deschner JB Hachulla E Leslie KS Mouy R Ferreira A Lheritier K Patel N Preiss R Hawkins PN Sustained remission of symptoms and improved health-related quality of life in patients with cryopyrin-associated periodic syndrome treated with canakinumab: results of a double-blind placebo-controlled randomized withdrawal study Arthritis Res Ther 2011 16 R202 10.1186/ar3535 22152723 PMC3334655

[B40] Berenbaum F Eymard F Houard X Osteoarthritis, inflammation and obesity Curr Opin Rheumatol 2013 16 114 118 10.1097/BOR.0b013e32835a9414 23090672

[B41] Fukuhara A Matsuda M Nishizawa M Segawa K Tanaka M Kishimoto K Matsuki Y Murakami M Ichisaka T Murakami H Ichisaka T Murakami H Watanabe E Takagi T Akiyoshi M Ohtsubo T Kihara S Yamashita S Makishima M Funahashi T Yamanaka S Hiramatsu R Matsuzawa Y Shimomura I Visfatin: a protein secreted by visceral fat that mimics the effects of insulin Science 2005 16 426 430 10.1126/science.1097243 15604363

[B42] Duan Y Hao D Li M Wu Z Li D Yang X Qiu G Increased synovial fluid visfatin is positively linked to cartilage degradation biomarkers in osteoarthritis Rheumatol Int 2012 16 1433 1437 10.1007/s00296-010-1731-8 21246369

[B43] Cirillo P Di Palma V Maresca F Pacifico F Ziviello F Bevilacqua M Trimarco B Leonardi A Chiariello M The adipokine visfatin induces tissue factor expression in human coronary artery endothelial cells: another piece in the adipokines puzzle Thromb Res 2012 16 403 408 10.1016/j.thromres.2012.06.007 22726553

[B44] Fan Y Meng S Wang Y Cao J Wang C Visfatin/PBEF/Nampt induces EMMPRIN and MMP-9 production in macrophages via the NAMPT-MAPK (p38, ERK1/2)-NF-kappaB signaling pathway Int J Mol Med 2011 16 607 615 21327328 10.3892/ijmm.2011.621

[B45] Romacho T Azcutia V Vazquez-Bella M Matesanz N Cercas E Nevado J Carraro R Rodriguez-Manas L Sanchez-Ferrer CF Peiro C Extracellular PBEF/NAMPT/visfatin activates pro-inflammatory signalling in human vascular smooth muscle cells through nicotinamide phosphoribosyltransferase activity Diabetologia 2009 16 2455 2463 10.1007/s00125-009-1509-2 19727662

[B46] Busso N Karababa M Nobile M Rolaz A Van Gool F Galli M Leo O So A De Smedt T Pharmacological inhibition of nicotinamide phosphoribosyltransferase/visfatin enzymatic activity identifies a new inflammatory pathway linked to NAD PLoS One 2008 16 e2267 10.1371/journal.pone.0002267 18493620 PMC2377336

[B47] Evans L Williams AS Hayes AJ Jones SA Nowell M Selective inhibition of PBEF/Visfatin/NAMPT suppresses leukocyte infiltration and cartilage degradation Arthritis Rheum 2011 16 1866 1877 10.1002/art.30338 21400478

[B48] Grahnert A Grahnert A Klein C Schilling E Wehrhahn J Hauschildt S Review: NAD +: a modulator of immune functions Innate Immun 2010 16 212 233 20388721 10.1177/1753425910361989

[B49] Koch-Nolte F Fischer S Haag F Ziegler M Compartmentation of NAD + -dependent signalling FEBS Lett 2011 16 1651 1656 10.1016/j.febslet.2011.03.045 21443875

[B50] Chang EJ Im YS Kay EP Kim JY Lee JE Lee HK The role of nerve growth factor in hyperosmolar stress induced apoptosis J Cell Physiol 2008 16 69 77 10.1002/jcp.21377 18300262

[B51] Friedman WJ Altiok N Fredholm BB Persson H Mechanisms of nerve growth factor mRNA regulation by interleukin-1 beta in hippocampal cultures: role of second messengers J Neurosci Res 1992 16 37 46 10.1002/jnr.490330106 1333537

[B52] Gosset M Berenbaum F Levy A Pigenet A Thirion S Cavadias S Jacques C Mechanical stress and prostaglandin E2 synthesis in cartilage Biorheology 2008 16 301 320 18836232

[B53] Berenbaum F Jacques C Thomas G Corvol MT Bereziat G Masliah J Synergistic effect of interleukin-1 beta and tumor necrosis factor alpha on PGE2 production by articular chondrocytes does not involve PLA2 stimulation Exp Cell Res 1996 16 379 384 10.1006/excr.1996.0047 8598226

[B54] Toyomoto M Ohta M Okumura K Yano H Matsumoto K Inoue S Hayashi K Ikeda K Prostaglandins are powerful inducers of NGF and BDNF production in mouse astrocyte cultures FEBS Lett 2004 16 211 215 10.1016/S0014-5793(04)00246-7 15044028

[B55] Dal Toso R De Bernardi MA Brooker G Costa E Mocchetti I Beta adrenergic and prostaglandin receptor activation increases nerve growth factor mRNA content in C6-2B rat astrocytoma cells J Pharmacol Exp Ther 1988 16 1190 1193 2458446

[B56] Chidiac JJ Al-Asmar B Rifai K Jabbur SJ Saade NE Inflammatory mediators released following application of irritants on the rat injured incisors, The effect of treatment with anti-inflammatory drugs Cytokine 2009 16 194 200 10.1016/j.cyto.2009.01.008 19261487

[B57] Samad TA Moore KA Sapirstein A Billet S Allchorne A Poole S Bonventre JV Woolf CJ Interleukin-1beta-mediated induction of Cox-2 in the CNS contributes to inflammatory pain hypersensitivity Nature 2001 16 471 475 10.1038/35068566 11260714

[B58] Bullo M Peeraully MR Trayhurn P Stimulation of NGF expression and secretion in 3 T3-L1 adipocytes by prostaglandins PGD2, PGJ2, and Delta12-PGJ2 Am J Physiol Endocrinol Metab 2005 16 E62 E67 10.1152/ajpendo.00008.2005 15713689

[B59] Borglum JD Pedersen SB Ailhaud G Negrel R Richelsen B Differential expression of prostaglandin receptor mRNAs during adipose cell differentiation Prostaglandins Other Lipid Mediat 1999 16 305 317 10.1016/S0090-6980(98)00082-3 10480485

[B60] Clark CA Schwarz EM Zhang X Ziran NM Drissi H O'Keefe RJ Zuscik MJ Differential regulation of EP receptor isoforms during chondrogenesis and chondrocyte maturation Biochem Biophys Res Commun 2005 16 764 776 10.1016/j.bbrc.2004.11.074 15694412

[B61] de Brum-Fernandes AJ Morisset S Bkaily G Patry C Characterization of the PGE2 receptor subtype in bovine chondrocytes in culture Br J Pharmacol 1996 16 1597 1604 10.1111/j.1476-5381.1996.tb15580.x 8842420 PMC1909846

