# Supplementary figures and images for: Induction of nerve growth factor expression and release by mechanical and inflammatory stimuli in chondrocytes: possible involvement in osteoarthritis pain
Source: Arthritis Res Ther. 2014 Jan 20;16(1):R16. doi: 10.1186/ar4443 (PMC3978639; doi:10.1186/ar4443)

**A**

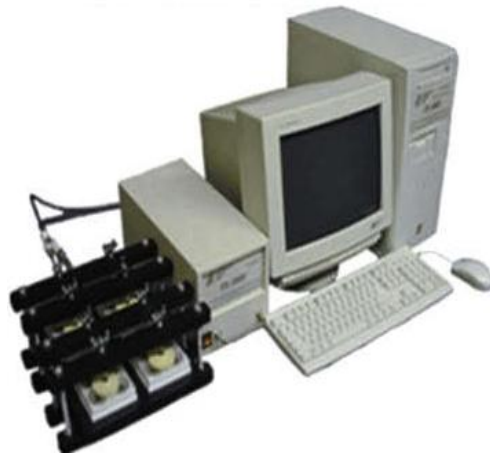

Flexercell Compression  
Plus System FX-4000C

**B**

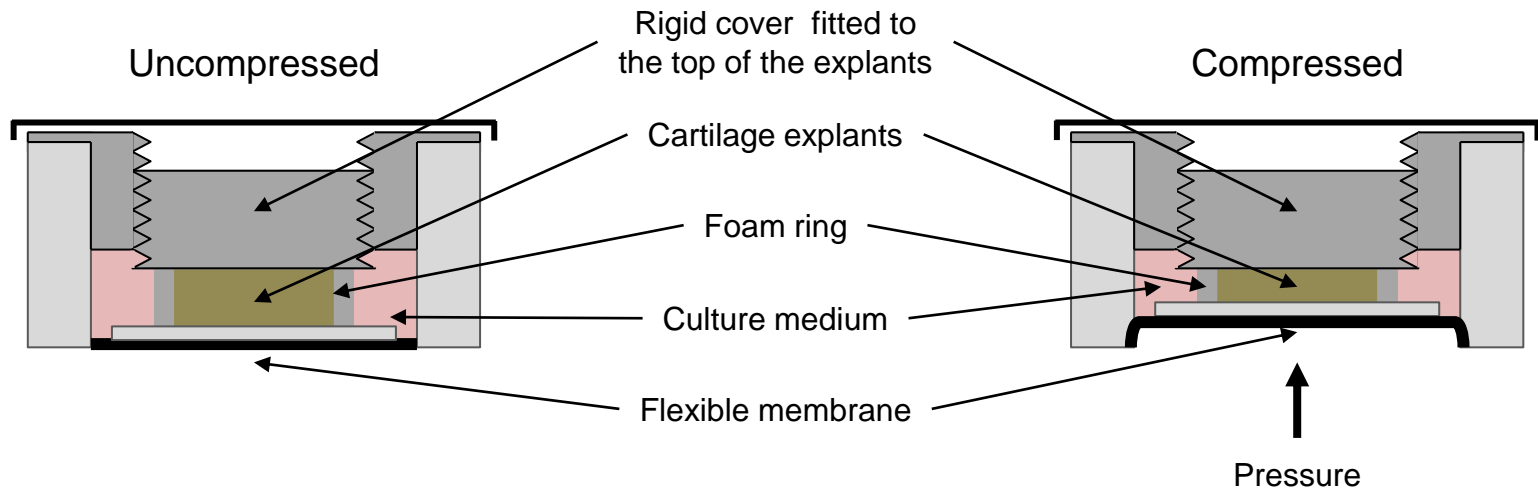

**Supplemental Figure**

Supplement: Additional file 1: Figure S1 — Compression system. (A) FX-4000C™ Flexercell™ Compression Plus™ System (Flexcell International Corp., Hillsborough, NC, USA). A positive pressure compresses samples between a piston and stationary platen on the BioPress™ culture. (B) Schematic diagram of the BioPress™ culture plate compression chamber in uncompressed or compressed position. [file ar4443-S1.pdf]
